# Polymerase mutations underlie early adaptation of H5N1 influenza virus to dairy cattle and other mammals

**DOI:** 10.1038/s41467-026-68306-6

**Published:** 2026-01-16

**Authors:** Vidhi Dholakia, Jessica L. Quantrill, Samuel A. S. Richardson, Nunticha Pankaew, Maryn D. Brown, Jiayun Yang, Fernando Capelastegui, Tereza Masonou, Katie-Marie Case, Jila Ajeian, Maximillian N. J. Woodall, Callum Magill, Graham Freimanis, Amy McCarron, Ecco Staller, Carol M. Sheppard, Ian H. Brown, Pablo R. Murcia, Claire M. Smith, Munir Iqbal, Paul Digard, Wendy S. Barclay, Rute M. Pinto, Thomas P. Peacock, Daniel H. Goldhill

**Affiliations:** 1 Department of Pathobiology and Population Sciences, Royal Veterinary College, London, UK; 2 Department of Infectious Disease, Imperial College London, London, UK; 3 The Pirbright Institute, Woking, UK; 4 The Roslin Institute, University of Edinburgh, Edinburgh, UK; 5 Great Ormond Street UCL Institute of Child Health, London, UK; 6 MRC—University of Glasgow Centre for Virus Research, Glasgow, Scotland UK; 7 Sir William Dunn School of Pathology, The University of Oxford, Oxford, UK

**Keywords:** Influenza virus, Virus-host interactions, Molecular evolution, Viral evolution

## Abstract

In 2024, an unprecedented outbreak of H5N1 high pathogenicity avian influenza was detected in dairy cattle in the USA resulting in spillbacks into poultry, wild birds and other mammals including humans. Here, we present molecular and virological evidence that the cattle B3.13 genotype H5N1 viruses rapidly accumulated adaptations in polymerase genes that enabled better replication in bovine cells and tissues, as well as cells of other mammals including humans. We find evidence of several mammalian adaptations in cattle including PB2 M631L, which is found in all cattle sequences, and PA K497R, which is found in the majority. Structurally, PB2 M631L maps to the polymerase-ANP32 interface, an essential host factor for viral genome replication. We show that this mutation adapts the polymerase to better interact with bovine ANP32 proteins, particularly ANP32A, and thereby enhances virus replication in bovine mammary systems and primary human airway cultures. We show that ongoing evolution in the PB2 gene, including E627K and a convergently arising D740N substitution, further increase polymerase activity and virus replication in a range of mammalian cells. Thus, circulation of H5N1 in dairy cattle allows virus adaption improving replicative ability in cattle and poses a continued risk of zoonotic spillover.

## Abstract (web summary)

Avian influenza jumped from wild birds into dairy cattle. Here, the authors report that two mutations in the viral polymerase helped the virus to quickly adapt to cattle. Mutations increased the polymerase activity and made the virus better at replicating in human cells.

## Introduction

The natural host reservoirs of influenza A viruses (IAV) are wild waterfowl and shorebirds. However, IAV can spill over and adapt to infect mammalian species, such as pigs, humans, horses and dogs^1^. In 2020, a novel genotype of H5N1 clade 2.3.4.4b emerged in Europe and spread around the world, causing a panzootic in birds^2^. This panzootic genotype and its progeny are notable for their frequent spillover into mammals, including confirmed or potential mammal-to-mammal spread^3^ in farmed mink in Europe^4–6^ (2022–2023), semi-aquatic marine mammals in South America and Antarctica^7–11^ (2023−), and recently, dairy cattle in the USA^12,13^ (2024−).

In early 2024, an unexplained drop in milk production in cattle in the USA was attributed to infection with H5N1 clade 2.3.4.4b, of the B3.13 genotype^12,13^. Infected cows were initially observed in Texas, but as of June 2025, over 1000 infected herds have been recorded across 17 US states^12,13^. This outbreak was most likely caused by a single spillover from wild birds^12–14^, with continuous transmission between dairy cattle (hereafter referred to as ‘cattle’) mediated by human activities such as contamination of milking machinery within farms^15^ and movement of infected animals and/or contaminated equipment between farms and states^13^. The virus does not cause high mortality in cattle; instead, it mostly causes mastitis and modest respiratory distress^12,16^. In addition to cattle, several other species have been infected following direct or indirect exposure to infected cattle, including chickens, turkeys, peridomestic birds, farmed alpaca, domestic cats^13^, and humans^17,18^. Infections have been repeatedly fatal in cats that ingested infected milk, but in humans, the B3.13 virus has been associated with conjunctivitis and/or mild respiratory disease^17,18^.

Avian influenza viruses (AIVs) usually have restricted replication in mammalian hosts^1^. However, they can rapidly accumulate mammalian adaptations that overcome this block, often in the viral RNA-dependent RNA polymerase^3,19,20^. In particular, ANP32 proteins are essential host-encoded factors co-opted by the virus to support genome replication^21,22^. All birds, excluding palaeognaths (e.g., ostriches and related birds), have an ANP32A gene with an additional exon resulting in splice variants encoding proteins with an extra 33 amino acids relative to mammalian orthologues^23^. Avian-origin influenza viruses require polymerase mutations to utilise the shorter mammalian ANP32A/B proteins^24^. The most widespread and best characterised adaptive mutations are those in the PB2 subunit of the polymerase, including PB2 E627K^23^, Q591R/K^25,26^, and D701N^26,27^, all of which have been detected during other mammalian H5N1 outbreaks^5–11,28,29^ but are absent in the initial dairy cattle viruses. However, alternative adaptive mutations in the influenza polymerase that enable the virus to co-opt suboptimal ANP32 proteins are possible^30–32^. Here, we investigated potential mammalian adaptations in the polymerase of cattle-adapted H5N1 viruses and identified two key mutations that allow the B3.13 virus to replicate in cells and tissues from cattle and other mammalian species, including humans.

## Results

### Phylogenetic analysis of the cattle H5N1 polymerase identified potential adaptive mutations

**Fig. 1:**
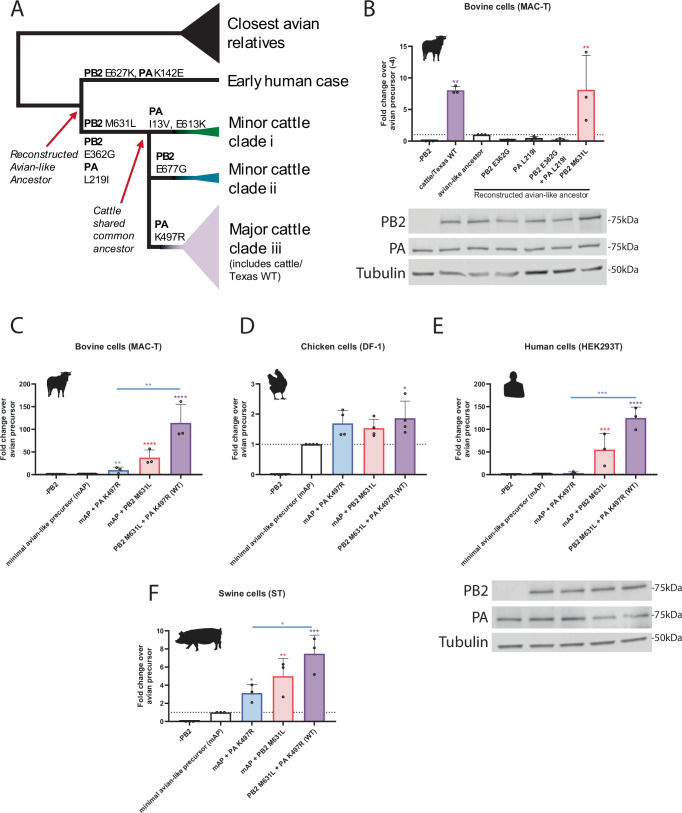
Identification of key mammalian polymerase adaptations in the major clade of bovine H5N1 that enhance polymerase activity in bovine cells. **A** Simplified schematic of cattle polymerase phylogeny and polymerase mutations that arose in early branches. **B**–**F** Minigenome in (**B**, **C**) bovine MAC-T cells (**D**) chicken DF-1 (**E**) human HEK293T, (**F**) swine ST cells with cattle Texas (WT). **B** Reconstructed avian-like ancestor has a reversion of PB2 E362G, M631L and PA L219I, K497R. **C**–**F** Minimal avian-like precursor has the reversions of PB2 M631L and PA K497R. Data normalised to a minimal avian-like precursor. Data throughout plotted as the mean of *N* = 3 independent repeats. **B**, **E** Expression of PB2 and PA was measured by western blot; tubulin was used as a loading control. Data throughout plotted as mean + SD. Statistics throughout performed by one-way ANOVA with multiple comparisons, comparing all data using log-transformed data. **B** Significance is only plotted if increased over the avian-like ancestor. Log-normality determined by the Shapiro-Wilk test and the QQ plot. Significance shown by asterisks indicating: *, 0.05  ≥  *P*  >  0.01; **, 0.01  ≥  *P*  >  0.001; ***, 0.001  ≥  *P*  >  0.0001; ****, *P*  ≤  0.0001. (B: cattle/Texas WT vs. avian-like ancestor *p* = 0.0023; PB2 M631L vs. avian-like ancestor *p* = 0.0043. C: mAP vs. mAP + PA K497R *P* = 0.0098; mAP vs. mAP + PB2 M631L *P* = 0.0003; mAP vs. PB2 M631L + PA K497R (WT) *P* < 0.0001; mAP + PA K497R vs WT *P* = 0.0018. D: mAP vs. WT *P* = 0.02; E: mAP vs. mAP + PB2 M631L *P* = 0.0002; mAP vs. WT *P* < 0.0001; mAP + PA K497R vs WT *P* = 0.0002. F: mAP vs. mAP + PA K497R *P* = 0.0133; mAP vs. mAP + PB2 M631L *P* = 0.0018; mAP vs. WT *P* = 0.0003; mAP + PA K497R vs WT *P* = 0.0426).

To understand how an avian influenza virus had adapted to efficiently replicate in cells of a novel mammalian species, we constructed a phylogeny using concatenated genomes of all influenza A viruses isolated from cattle, the virus from the first reported human case and wild avian viruses from September 2023 to March 2024 (Supplementary Fig. S1). Viruses from cattle formed a monophyletic group suggesting a single spillover event from wild birds^13,14^ (Fig. 1A). The sequence of the first case of human infection with a B3.13 H5N1 virus^17^ was an outlier relative to all subsequent cattle B3.13 viruses (Fig. 1A, Supplementary Fig. S1) and contained the known mammalian adaptations PB2 E627K^33^ as well as PA K142E^34^ relative to the closest avian relatives (Supplementary Fig. S1). However, these adaptive mutations were not found in any cattle virus sequences, which instead all contained PB2 M631L as well as PB2 E362G and PA L219I. PB2 M631L has previously been reported to adapt avian H1N1, H5N1 and H10N7 viruses to mammals^33,35,36^, as well as H9N2 viruses to gene-edited chickens with a non-functional ANP32A^32^. The phylogeny strongly suggests that M631L evolved in cattle, although there is a small possibility that it could have arisen in birds prior to transmission into cattle^14^. Early cattle sequences grouped into three clades with additional mutations in the polymerase segments including i) PB2 E677G^37,38^, ii) PA I13V and E613K, and iii) PA K497R, which was found in the largest cattle clade (Fig. 1A, Supplementary Fig. S1), consisting of ~95% of sequences (as of December 2024) and has previously been associated with influenza adaptation to mammals^39^. Minor clade i (PB2 E677G) was found solely in Texas and appeared only at the beginning of the outbreak whereas minor clade ii (PA I13V) was detected across multiple states, including, most recently, in Colorado.

### Activity of bovine H5N1 polymerase in mammalian cells maps to PB2 M631L and PA K497R

To test whether the mutations identified from the phylogenetic analysis were adaptive in cattle, we first constructed a ‘minireplicon’ system suitable for testing AIV polymerase activity in bovine cells. This consisted of co-transfecting bovine cells with a custom-made minigenome reporter that uses a bovine RNA polymerase I (Pol I) promoter to express a viral-like RNA encoding firefly luciferase flanked with non-coding regions from influenza A segment 8/NS^40^, along with plasmids expressing influenza virus ribonucleoprotein components, PB2, PB1, PA and nucleoprotein (NP). This species-specific minireplicon system showed 10–20-fold higher activity in bovine cells than one using an equivalent human PolI reporter (Supplementary Fig. S2). Using this bovine-specific minireplicon reporter, we showed that the reconstituted polymerase complex of A/dairy cattle/Texas/24-008749-001-original/2024 (cattle/Texas) was highly active in MAC-T bovine mammary epithelial cells^41^ (Fig. 1B).

We next reconstructed an avian-like ancestor to represent the polymerase complex of the virus that most likely entered the cattle population, by reverting potential adaptive mutations from the PB2 and PA segments of cattle/Texas: PB2 M631L, PB2 E362G, PA K497R and PA L219I (Fig. 1A, Supplementary Fig. S1). Whilst still active above background, the avian-like ancestral polymerase was much less active than its WT cattle/Texas counterpart (Fig. 1B). To begin to identify key changes, we assessed the impact of adding back the mutations that arose between this reconstructed avian-like ancestor and the cattle virus shared common ancestor (PB2 M631L, PB2 E362G and PA L219I). Neither PB2 E362G nor PA L219I increased polymerase activity in bovine cells relative to the avian-like ancestor background, either individually or in combination, and PB2 E362G even reduced activity (Fig. 1B). In contrast, PB2 M631L increased activity by around 10-fold, suggesting this was the key polymerase mutation that arose early in the adaptation of H5N1 to dairy cattle.

We also investigated later mutations that arose in the major clade III of the cattle H5N1 virus (Fig. 1A). We first tested the effect of the PA mutation K497R that is maintained across all sequences of the major bovine clade. Reverting both PB2 631 L to M and PA 497 R to K (generating a ‘minimal avian-like precursor’ or mAP) resulted in a ~ 100-fold decrease in polymerase activity in bovine cells (Fig. 1C). The reintroduction of PB2 M631L alone increased the signal by around 50-fold. Reintroducing PA K497R had a more modest effect (~10-fold increase in signal). Neither mutation alone fully recapitulated the activity of the WT polymerase, indicating that the PA change was further adaptive. The same mutations had minimal impacts (<2-fold) in avian cells (chicken fibroblasts, DF-1; Fig. 1D), consistent with an adaptive function in cattle cells rather than simply altering the intrinsic activity of the viral polymerase.

We next tested the impact of these two mutations on polymerase activity in cells from two other mammalian hosts relevant to influenza viruses—humans and swine, again using host species-specific minireplicon reporters (Fig. 1E, F). As seen in the bovine cells, both adaptations individually significantly enhanced polymerase activity, with PB2 M631L having the greater effect, suggesting it is the major mammalian adaptation across different mammalian species. Also, as observed in bovine cells, we noted a cumulative effect of the two substitutions together.

To investigate whether PB2 M631L and PA K497R could be universal mammalian adaptations for H5 viruses, we introduced them into other H5N1 polymerase backgrounds: a recent 2.3.4.4b virus (AIV07- A/chicken/England/053052/2021), an EU reference laboratory (EURL) genotype C common in the UK in 2021/22 and a distantly related H5N1 IAV polymerase (A/turkey/England/50–92/1991; 50–92), both of which originated from low pathogenicity avian influenza virus donors^42^. Relative to cattle/Texas, AIV07 and 50–92 have amino acid identities for PB2 of 98% and for PA of 98% and 97%, respectively. In these additional genetic backgrounds, both PB2 M631L and PA K497R significantly enhanced polymerase activity in bovine cells and human cells (Supplementary Fig. S3A–F). When compared to the well-characterised mammalian adaptation PB2 E627K, we saw that neither individual mutation increased polymerase activity to the level of E627K, but when combined, there was no statistical difference seen between PB2 M631L/PA K497R and E627K, suggesting the combined mutations are just as potent. There was, however, a trend that E627K alone had a larger effect relative to PB2 M631L + PA K497R in the human cells compared to the cattle cells. Overall, this suggests both PB2 M631L and PA K497R are bona fide mammalian adaptations, relevant across diverse AIVs.

### PB2 M631L, and to a lesser extent PA K497R, enable efficient replication of bovine H5N1 in mammalian cells and explants

**Fig. 2:**
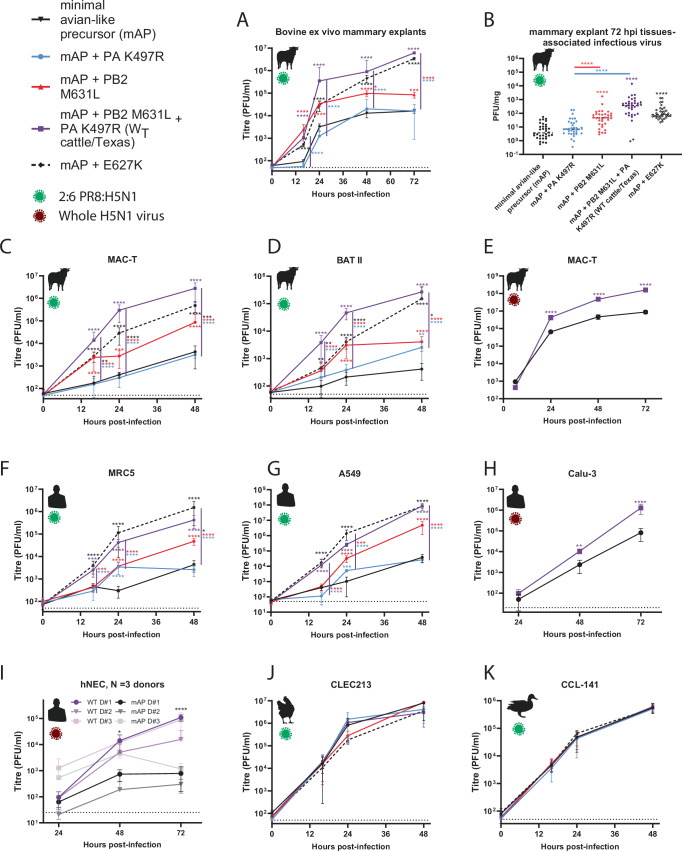
PB2 M631L, and to a lesser extent PA K497R, enhance virus replication in mammalian, but not avian culture systems. Reverse-genetics derived viruses, containing the HA and NA genes of the attenuated laboratory strain A/Puerto Rico/8/34, and the remaining genes from Cattle Texas (2:6 viruses; panels (**A**–**D**, **F**, **G**, **J**, **K**)) or reverse genetics derived full H5N1 viruses (whole virus, panels E, H and I) were used to infect **A**, **B** bovine ex vivo mammary explants, **C**, **E** bovine mammary cells (MAC-T), **D** bovine respiratory cells (BAT II) cells, **F** human lung cells (MRC5), **G** human lung adenocarcinoma cells (A549), **H** human lung cells (Calu-3), **I** primary human nasal epithelial cultures (hNECs) maintained at air liquid interface. **J** chicken lung cells (CLEC213) or **K** duck fibroblasts (CCL-441). Explants were infected with 5000 pfu/explant. Cell lines were infected at an MOI of 0.01. Infectious virus titres were determined by plaque assay on MDCKs. Data from bovine explants represents *N* = 34 repeats from 3 donor cattle from 3 different independent experiments. Data from cell lines plotted as (**C**), 2 x *N* = 4, (**D**, **F**, **G**, **J**, K) 2 x *N* = 3 technical repeats, (**E**, **H**) *N* = 3 repeats, from a representative repeat of *N* = 3 independent repeats and I) 3 independent donors with virus replication tested in triplicate. Data throughout plotted as mean ± SD. Statistics throughout performed by two-way ANOVA on log-transformed data with multiple comparisons between all data points within a single time point. Significance was only plotted when differences were significant to the minimal avian precursor (shown as matched colour asterisks), or between the WT cattle/Texas and mutants (shown as coloured asterisks next to the purple line). Significance shown by asterisks indicating: *, 0.05  ≥  *P*  >  0.01; **, 0.01  ≥  *P*  >  0.001; ***, 0.001  ≥  *P*  >  0.0001; ****, *P*  ≤  0.0001. Where *P* ≥ 0.0001, exact *P*-values A: mAP + PB2 M631L vs WT at 48 h, *P* = 0.0381, mAP vs mAP + PB2 M631L at 72 h *P* = 0.0001. C: WT vs mAP + E627K 16 h *P* = 0.006, 48 h *P* = 0.0004; mAP vs mAP + PB2 M631L 24 h *P* = 0.0001. D: mAP vs mAP + E627K 16 h *P* = 0.0061; WT vs mAP + E627K 16 h *P* = 0.0001, 48 h *P* = 0.0378; mAP vs mAP + PA K497R 48 h *P* = 0.0036. F: mAP vs WT 16 h *P* = 0.0008; mAP + PB2 M631L vs WT 16 h *P* = 0.0003; WT vs mAP + E627K 48 h *P* = 0.0157. G: mAP vs mAP + K497R 16 h *P* = 0.0411, 24 h *P* = 0.0007; mAP + M631L vs WT 24 h *P* = 0.0001. H: 48 h *P* = 0.0029. I: All WT vs All mAP 48 h *P* = 0.0197.

To further test the importance of PB2 M631L and PA K497R in adapting the virus to replicate in mammalian cells, we generated a reverse genetics system for cattle/Texas and made viruses bearing mutations at these sites. To work safely at BSL2, we rescued viruses as 2:6 reassortants containing the HA and NA of the attenuated vaccine strain, A/Puerto Rico/8/1934 (PR8)^43^ and the remaining genes from cattle/Texas. We also conducted work with full reverse genetics-derived viruses at ACDP3/SAPO4 (BSL3+). We compared multicycle replication of the WT cattle/Texas virus to either the individual or double PB2/PA reversions in bovine ex vivo mammary gland explants (Fig. 2A). Compared to the minimal avian-like precursor (WT cattle/Texas virus with PB2 M631L and PA K497R reverted), the viruses with PB2 M631L alone or in combination with PA K497R showed a significant, >10-fold increase in infectious virus titres at 24 and 48 h. However, PB2 M631L alone grew to significantly lower titres than the WT virus that contained K497R as well at 48 and 72 h post-infection (Fig. 2A). This was reflected both in released virus from the explants, as well as tissue-associated virus harvested at 72 h post-infection (Fig. 2B).

Consistent results were also seen in MAC-T cells and bovine alveolar cells (BAT II; Fig. 2C–E), where PB2 M631L, but not PA K497R alone, significantly enhanced replication across multiple tested time points. Again, PB2 M631L replicated to significantly lower titres than the wild-type virus, also containing PA K497R. Overall, these results in the context of infectious virus are consistent with the minireplicon data that show PB2 M631L, and to a lesser degree PA K497R, are responsible for this virus’s ability to replicate in bovine cells.

We next tested the replication of these viruses in several human lung cell lines (MRC5, A549 and Calu3; Fig. 2F–H). Consistent with the minireplicon assays in human cells (Fig. 1E) and virus replication phenotypes in bovine cells (Fig. 2A–E), the PB2 M631L mutation alone, or combined with PA K497R, significantly enhanced virus titres compared to the minimal avian-like precursor virus across multiple time points in the human cell lines. While PA K497R alone showed only minor effects, PB2 M631L alone replicated to significantly lower titres than the WT virus (cattle/Texas), again showing PA K497R, in particular, boosted replication when paired with PB2 M631L. We also tested the replication of the WT and mAP virus in primary human nasal epithelial cultures (hNECs) from 3 adult donors and maintained at air-liquid interface (Fig. 2I). The mAP virus replicated to significantly lower titres than WT in all 3 donors at 72 h post-infection. Overall, these data further establish that PB2 M631L is a major mammalian adaptation that enhances virus replication in both bovine and human cells.

Next, we tested the growth of these viruses in a pair of avian cell lines (chicken CLEC213 and duck CCL-141; Fig. 2J, K). All mutants showed similar growth kinetics to the wild-type virus, suggesting that these mutations have a minimal impact on virus fitness/replication in avian cells.

Across the growth experiments in Fig. 2, we also compared the relative replication of the mAP and the mAP containing PB2 E627K. Consistently, across all bovine cell lines and explants (Fig. 2A–D), the combination of PB2 M631L and PA K497R resulted in significantly higher titres than mAP + E627K. Conversely, in the human cells (Fig. 2F, G), the mAP + E627K virus replicated to higher titres (albeit not significantly) than the WT cattle virus. Overall, this might suggest why PB2 M631L and PA K497R were favoured in the cattle virus, whereas PB2 E627K is more common in human infections.

All available H5N1 sequences from this cattle outbreak contain PB2 M631L, but PA K497R is only found in the major clade of cattle sequences (clade iii). Minor clade i and ii viruses contain alternative PB2 or PA mutations (Fig. 1A, Supplementary Fig. 1). To investigate whether these minor clade mutations represent alternative bovine adaptations, we recapitulated these genotypes by individually introducing PA I13V and E613K from clade i or PB2 E677G from clade ii into the cattle/Texas polymerase background with PA R497 reverted to the avian consensus 497 K. PA I13V and E613K together exerted a small but significant increase in polymerase activity over PB2 M631L alone, showing a ~ 3-fold increased minireplicon signal in bovine cells (Supplementary Fig. S4A) and human cells (Supplementary Fig. S4B). The introduction of PB2 E677G had no impact on bovine cells but resulted in a significant ~4-fold increase in polymerase activity in human cells (Supplementary Fig. S4B). The polymerase mutations found in the minor clade i and ii cattle viruses had at most a very minor impact (<1.5-fold) in polymerase activity in avian cells (Supplementary Fig. S4C). Overall, this suggests that PB2 M631L was the initial potent mammalian adaptation, and a range of other polymerase mutations then ‘fine-tuned’ polymerase activity in bovine cells.

### More recent mutations in cattle sublineages further enhance B3.13 H5N1 polymerase activity in mammalian but not avian cells

**Fig. 3:**
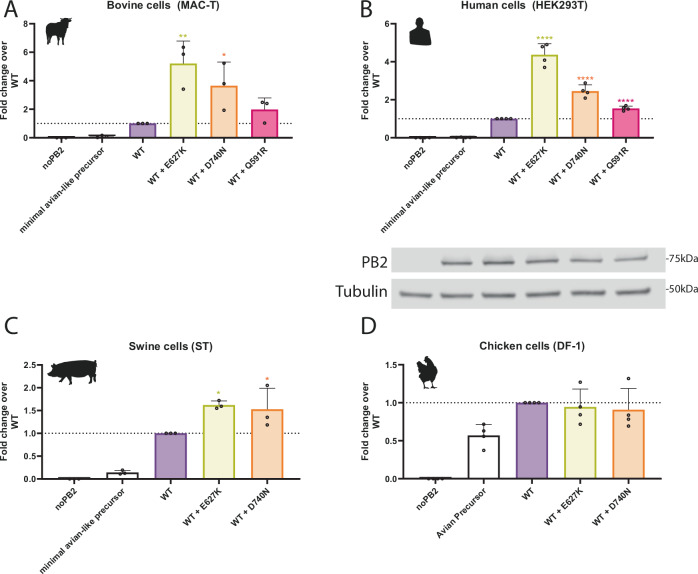
Ongoing adaptations found in cattle H5N1 viruses further enhance polymerase activity in mammalian cells. Minigenome in **A** bovine MAC-T cells, **B** human HEK293T, **C** swine ST cells or **D** chicken DF-1 with mutant versions of cattle Texas representing potential ongoing adaptation. Data normalised to WT cattle, Texas. Data throughout plotted as the mean of *N* = 3 independent biological repeats. **B** Matched western blot showing PB2 expression. Data throughout plotted as mean + SD. Statistics throughout performed by one-way ANOVA with multiple comparisons against WT polymerase using log-transformed data. Log-normality determined by the Shapiro-Wilk test and QQ plot. Significance shown by asterisks indicating: *, 0.05  ≥  *P*  >  0.01; **, 0.01  ≥  *P*  >  0.001; ***, 0.001  ≥  *P*  >  0.0001; ****, *P*  ≤  0.0001. (A: WT vs WT + E627K *P* = 0.0027; WT vs WT + D740N *P* = 0.0137. B: All **** *P*  ≤  0.0001. C: WT vs WT + E627K *P* = 0.0227; WT vs WT + D740N *P* = 0.0491).

By continually monitoring sequences from the ongoing dairy cattle outbreak, we detected incidences of further, known mammalian adaptations arising within polymerase genes of the cattle viruses^26^. This included a single emergence of a cluster of sequences containing PB2 Q591R, at least three independent emergences of PB2 D740N across the tree (Supplementary Fig. S1), as well as a more recent cluster from 2025 containing PB2 E627K. To test the impact of these changes, we introduced them into the WT cattle/Texas polymerase. PB2 D740N significantly boosted polymerase activity in bovine (~4-fold increased signal), human (~2-fold increase signal) and swine cells (1.2-fold increase; Fig. 3A–C). PB2 Q591R led to a smaller impact, reaching statistical significance in the human cells (Fig. 3A, B). The introduction of PB2 E627K on top of M631L consistently led to the highest polymerase activity (Fig. 3A–C). However, neither PB2 E627K nor D740N increased activity in avian cells, relative to the wild-type cattle/Texas polymerase (Fig. 3D). We conclude that these mutations indicate ongoing adaptation of H5N1 viruses to further optimise polymerase function in cattle.

**Fig. 4:**
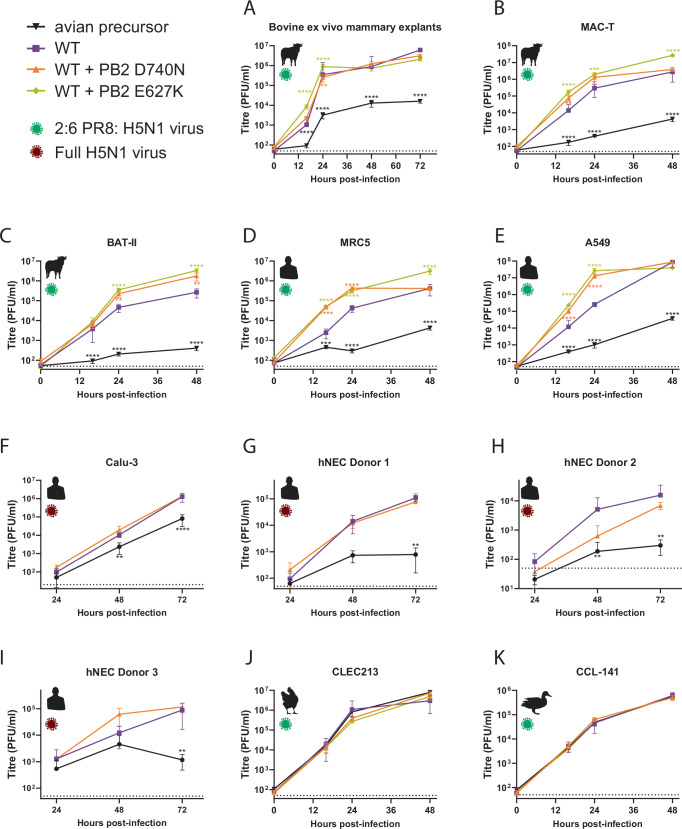
Further emerging mammalian adaptations in the cattle H5N1 PB2 enhance its replication in human and bovine, but not avian culture systems. Reverse-genetics derived viruses, containing the HA and NA genes of the attenuated laboratory strain A/Puerto Rico/8/34, and the remaining genes from Cattle Texas (2:6 viruses; panels, (**A**–**E**, **J**, **K**)) or reverse genetics derived full H5N1 viruses (whole virus, panels (**F**–**I**) were used to infect) **A** bovine ex vivo mammary explants, **B** bovine mammary cells (MAC-T), **C** bovine respiratory cells (BAT II) cells, **D** human lung cells (MRC5), **E** human lung adenocarcinoma cells (A549), **F** human lung cells (Calu-3), **G**–**I** primary differentiated human nasal epithelial cultures (hNECs) maintained at air liquid interface (hNECs), **J** chicken lung cells, or **K** duck fibroblasts (CCL-141). Cells were infected with an MOI of 0.01, and explants were infected with 5000 PFU/explant. Infectious virus titres were determined by plaque assay on MDCKs. **A** Data from bovine explants represents total *N* = 34 repeats from 3 donor cattle from 3 different independent experiments. Data from cell lines plotted as (**B**), 2 x *N* = 4, (**C**–**E**, **J**, **F**) 2 x *N* = 3 technical repeats, **F** *N* = 3 repeats, from a representative repeat of *N* = 3 independent repeats and **G**–**I** 3 independent donors with virus replication tested in triplicate. WT and minimal avian precursor data through the figure are the same shown through Fig. 2. Data throughout are plotted as mean ± SD. Statistics throughout performed by two-way ANOVA on log-transformed data with multiple comparisons against WT cattle/Texas. Significance shown by asterisks indicating: *, 0.05  ≥  *P*  >  0.01; **, 0.01  ≥  *P*  >  0.001; ***, 0.001  ≥  *P*  >  0.0001; ****, *P*  ≤  0.0001. (Where *P* ≥ 0.0001, exact *P*-values A: WT vs WT + PB2 D740N 24 h *P* = 0.0096. B: WT vs WT + PB2 D740N 16 h *P* = 0.0095, 24 h *P* = 0.0194; WT vs WT + PB2 E627K 24 h *P *= 0.0002. C: WT vs WT + PB2 D740N 24 h *P* = 0.0052, 48 h *P* = 0.0035. F: WT vs mAP 48 h *P* = 0.0029. G: WT vs mAP 72 h *P* = 0.0017. H: WT vs mAP 48 h *P* = 0.0040, 72 h *P* = 0.0017. I: WT vs mAP 72 h *P* = 0.0011).

To further investigate the impact of these ongoing mammalian adaptations, we rescued WT cattle/Texas + PB2 D740N. For work at CL2, we also rescued PR8:H5N1 2:6 reassortants of cattle/Texas with PB2 D740N or PB2 E627K. We initially tested the ability of the viruses to replicate in bovine mammary explants, and found that while PB2 D740N closely tracked the wild type virus, PB2 E627K reached significantly higher titres at early time points (Fig. 4A). Similar results were seen in MAC-T and BAT-II cells where both D740N and E627K significantly enhanced replication in the WT across multiple time points (Fig. 4B, C).

We also tested 2:6 virus in MRC5 and A549 cells (Fig. 4D, E) and found both D740N and E627K significantly increased virus titres across multiple time points. In a human lung cell line (Calu-3), using the full H5N1 virus, D740N trended towards higher, albeit but non-significantly higher titres than WT virus (Fig. 4F). Finally, we performed multicycle replication curves with whole H5N1 virus in primary differentiated human nasal epithelial cultures (hNECs), maintained at air liquid interface, taken from three independent adult donors (Fig. 4G–I). PB2 D740N behaved variably between donors and did not reach titres significantly different from the WT virus. We performed whole genome sequencing on the H5N1 viruses from two of the donors at 72 h post-infection and found no evidence of adaptation or selection at the consensus level following virus replication. Analysis of polymorphisms (>4% frequency) revealed no mutations associated with mammalian adaptation in the HA, PB2 and PA segments in any sequenced samples (Supplementary Fig. S5).

Finally, we tested virus replication kinetics (in the 2:6 background) in chicken and duck cells (Fig. 4J, K). We saw no significant difference in virus replication kinetics.

The PB2 E627K virus showed consistently higher titres than the wild type, suggesting this mutation likely increases the ability to replicate in both human and bovine cells, while the PB2 D740N-containing viruses showed a variable phenotype, suggesting that if this mutation does impact the virus, the effect is subtle and not consistently captured by these assays. Both E627K and D740N also appear to have a neutral impact on the ability to replicate in avian cells, suggesting these mutations could be maintained if viruses spill back into avian species.

### Mutations in the polymerase of the cattle H5N1 viruses adapt the polymerase to use bovine ANP32 proteins

**Fig. 5:**
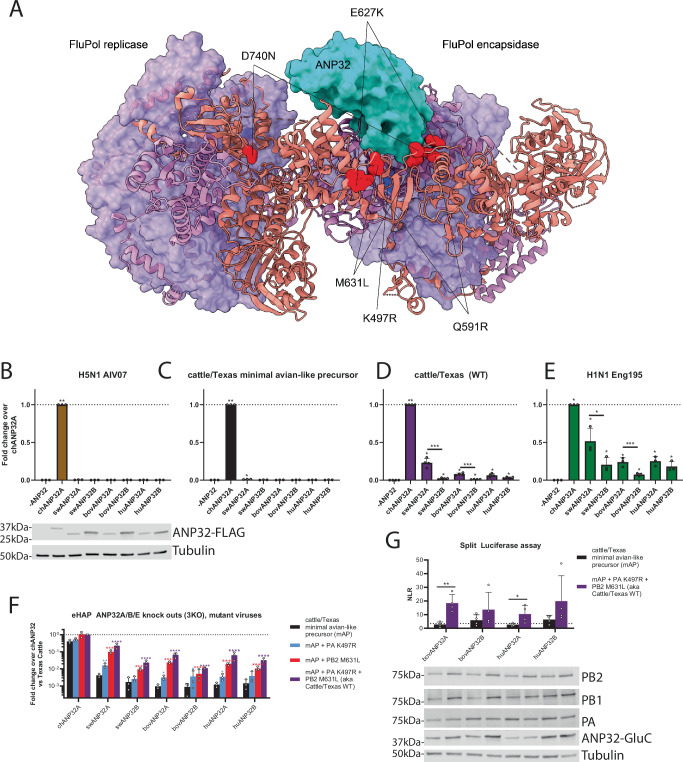
Although bovine ANP32A and B proteins are both able to support mammalian-adapted polymerases, bovine ANP32A is dominant which correlates with a stronger interaction. **A** Key mammalian adaptations identified in this study mapped to the structure of H5N1 influenza A replicase dimer complex with human ANP32B (PDB: 8R1J). ANP32B is shown in cyan, PB1 in purple, PA in violet and PB2 as a salmon ribbon representation. H5N1 cattle PB2 substitutions (Q591R, E627K, M631L and D740N) shown in red and PA (K497R) shown in blue. **B**–**F** Minigenomes in human engineered haploid cells (eHAP) with endogenous ANP32A, ANP32B and ANP32E knocked out were supplemented with ANP32 proteins from different species (chANP32A = chicken ANP32A, swANP32A = swine ANP32A, swANP32B = swine ANP32B, bovANP32A = bovine ANP32A, bovANP32B = bovine ANP32B, huANP32A = human ANP32A, huANP32B = human ANP32B, -ANP = no ANP32 control). Avianised cattle = PB2 M631L and PA K497R reverted. Data normalised throughout to chANP32A. **G** Split luciferase assays in 293 T cells showing the relative binding of different ANP32 proteins to the trimeric polymerase from cattle/Texas or the avianised version lacking PB2 M631L and PA K497R. PB1 was tagged with the N-terminal section of *Gaussia* luciferase, while the ANP32 proteins were tagged with the C-terminal portion. NLR, normalised luminescence ratios, were calculated from the ratio between signals between tagged and untagged ANP32/PB1 pairs. Dotted line equals a false positive cutoff of <2.5% as used in Mistry et al.^61^. **B**, **G** Matched expression of polymerase and ANP32 constructs shown by western blot. Data throughout plotted the mean of *N* = 3 (B-F) or *N* = 4 (**G**) independent biological repeats. Data throughout plotted as mean + SD. **B**–**E** Statistics performed by one-way ANOVA with multiple comparisons - against -ANP32 control, or between species-matched ANP32A and ANP32B proteins, using log-transformed data. **F** Statistics performed by two-way ANOVA with multiple comparisons comparing the different polymerase mutants within the same species, ANP32, on log-transformed data. **G** Statistics performed by multiple unpaired *T*-tests (2-tailed) between cattle/Texas WT and the minimal avian precursor. Log-normality determined by the Shapiro-Wilk test and the QQ plot. Significance shown by asterisks indicating: *, 0.05  ≥  *P*  >  0.01; **, 0.01  ≥  *P*  >  0.001; ***, 0.001  ≥  *P*  >  0.0001; ****, *P*  ≤  0.0001. (Where *P* ≥ 0.0001, exact *P*-values B: -ANP32 vs chANP32A *P* = 0.0035. C: -ANP32 vs chANP32A *P* = 0.0065; -ANP32 vs swANP32A *P* = 0.0454. D: -ANP32 vs chANP32A *P* = 0.0038; -ANP32 vs swANP32A *P* = 0.0101; -ANP32 vs swANP32B *P* = 0.0443; -ANP32 vs bovANP32A *P* = 0.0114; -ANP32 vs bovANP32B *P* = 0.0485; -ANP32 vs huANP32A *P* = 0.0218; -ANP32 vs huANP32B *P* = 0.0274; swANP32A vs swANP32B *P* = 0.0004; bovANP32A vs bovANP32B *P* = 0.0004. E: -ANP32 vs chANP32A *P* = 0.0163; -ANP32 vs swANP32A *P* = 0.0141; -ANP32 vs swANP32B *P* = 0.0127; -ANP32 vs bovANP32A *P* = 0.0158; -ANP32 vs bovANP32B *P* = 0.0229; -ANP32 vs huANP32A *P* = 0.0138; -ANP32 vs huANP32B *P* = 0.0166; swANP32A vs swANP32B *P* = 0.0299; bovANP32A vs bovANP32B *P* = 0.0004. F: swANP32A mAP vs mAP + PA K497R *P* = 0.0039; bovANP32A mAP vs mAP + PA K497R *P* = 0.0116; bovANP32B mAP vs mAP + PA K497R *P* = 0.0152; huvANP32A mAP vs mAP + PA K497R *P* = 0.0310. G: bovANP32A *p* = 0.0034; huANP32A *p* = 0.0491).

PB2 residue 631 is located close to the interface between influenza polymerase and the host factor ANP32 (Fig. 5A, Supplementary Figs. S6), near several other mammalian ANP32-adapting mutations such as PB2 E627K and Q591R^26,44^. We therefore hypothesised that the B3.13 polymerase adapted to bovine cells by increasing its ability to co-opt bovine ANP32 proteins. To test this, we performed minigenome assays in human cells, which lack ANP32A, ANP32B and ANP32E (eHAP triple knockout cells)^31^, complemented with ANP32 proteins from other relevant species including cattle, human, pigs, and chicken. Only chicken ANP32A robustly supported viral polymerases from avian (AIV07, an early European 2.3.4.4b panzootic genotype C virus; Fig. 5B) or avian-like viruses (the minimal avian-like precursor; Fig. 5C). Neither human or bovine ANP32A or B supported any significant activity of avian virus polymerases. In contrast, both human and bovine ANP32A/B supported mammalian-adapted polymerases: cattle/Texas (Fig. 5D) and a pandemic 2009 H1N1 virus (Fig. 5E), A/England/195/2009 (Eng195). However, bovine ANP32A was consistently and significantly better able than bovine ANP32B to support polymerase activity of all constellations tested. We also assessed the effect of the individual cattle/Texas mammalian adaptations, PB2 M631L and PA K497R on complementation by the different ANP32 homologues (Fig. 5F). Either mutation alone significantly enhanced polymerase activity supported by bovine ANP32A or ANP32B, with PB2 M631L showing a stronger effect with all ANP32 orthologues than PA K497R. Thus, we conclude that the major adaptive mutation PB2 M631L detected in the bovine influenza outbreak has adapted IAV for replication in cattle by increasing its capacity to co-opt bovine ANP32A.

Finally, to test the mechanism by which the bovine H5N1 virus was able to better use bovine ANP32, we performed a split-luciferase assay to test protein-protein interactions (Fig. 5G). We tagged the cattle/Texas PB1 with one half of a split *Gaussia* luciferase and tagged different ANP32 proteins with the other half of the split luciferase. We tested the ability of the minimal Avian Precursor or WT cattle/Texas polymerases to interact with bovine or human ANP32A or B. We found that the polymerase mutations in cattle/Texas significantly enhanced the ability of the polymerase to interact with both bovine and human ANP32A/B. A stronger interaction was seen with bovine ANP32A compared to ANP32B, suggesting that PB2 M631L and PA K497R specifically promote effective binding and use of bovine ANP32A protein.

## Discussion

Our data using reconstructed viral polymerases in host-specific minireplicon and infection assays in cells and explants from different relevant influenza host species show that PB2 M631L is the key adaptive mutation that allowed B3.13 H5N1 high pathogenicity avian influenza virus to efficiently replicate in cattle. PB2 M631L enables the polymerase complex to co-opt bovine ANP32 proteins to support its activity, with a strong preference for bovine ANP32A over ANP32B. This is likely due to bovine ANP32B containing an arginine at position 153, which we have previously shown is responsible for the reduced ability of canine ANP32B to support influenza polymerase^26^. Structurally, in the complex between the influenza polymerase and host ANP32, PB2 residue 631 is located near amino acids 627 and 591, which are well-characterised sites that mutate to allow adaptation of AIV polymerase to mammalian ANP32 proteins^26,44^. However, the exact mechanism of how PB2 M631L allows the use of mammalian ANP32s remains unresolved, although our data suggests a direct enhanced interaction. Structural studies show that ANP32 bridges the asymmetric dimer of influenza polymerase, comprising a replicating subunit and a recruited subunit that will be used to encapsidate the nascent RNA strand. PB2 E627K results in a switch from a negative to a positive charge, enhancing the interaction between the encapsidating subunit of the polymerase replication dimer and ANP32 residue E151, while also avoiding the clash between the negatively charged LCAR region of mammalian ANP32s and the replicating polymerase subunit^44,45^. This mechanism also likely applies to adaptive charge switch mutations at residue 591 from Q to R/K^44^. PB2 M631L does not result in an alteration of charge but presumably changes the polymerase structure in this region to better accommodate the interaction between either or both polymerase subunits and ANP32. Recent work from Gu et al. using the cattle virus polymerase confirmed that PB2 M631L increases activity in human cells at both 33 °C and 37 °C^33^. Previous work has shown that PB2 M631L adapts avian H10N7 viruses to mice^35^ and enables an avian H9N2 virus to use otherwise suboptimal ANP32 proteins^32,46^. These reports, combined with our results in the context of a second 2.3.4.4b genotype and an older non-goose/Guangdong H5N1, suggest that M631L is a general mammalian adaptive mutation for influenza polymerase that is not specific to a particular virus genetic background. However, why this mutation emerged in cattle rather than other pathways for adaptation including the more potent PB2 E627K remains unclear. We consistently found that when using the minimal Avian Precursor (cattle WT with PB2 M631L + PA K497R removed), the addition of E627K showed lower replication in bovine, but not human systems, than the combined addition of M631L and PA K497R. There was also similar or lower polymerase activity observed in the corresponding minigenome assays. Our previous work has shown that E627K is biased towards emerging in hosts or systems where the virus preferentially uses ANP32B^26^; however, in cattle, we found that ANP32A better supported the polymerase. E627K has arisen experimentally in cattle infected with a contemporary European H5N1 2.3.4.4b virus^16^.

Following fixation of PB2 M631L in cattle, additional mutations in the polymerase arose in three separate clades. Most cattle sequences contain PA K497R, which increased polymerase activity in the absence of M631L and virus replication in bovine mammary cells. Structurally, PA K497R sits in a loop close to the PB2 M631L and ANP32 interface, though it may not be directly interacting with them (Fig. 5A). Additionally, PA K497R sits at the FluPol symmetric dimer interface and therefore may modulate the dynamics of dimer formation in the same manner as the mammalian adaptation PA Q556R^31^. Of the mutations defining the other two early cattle virus clades, the combination of PA I13V + E613K or PB2 E677G also showed modest increases in activity, although neither group spread particularly widely. Neither PA I13V or E613K have previously been associated with mammalian adaptation but E613K does fall on the polymerase asymmetric dimer interface (Supplementary Fig. S6B) and could be altering polymerase regulation^44^.

Cattle/Texas virus has extremely robust replication in the bovine mammary gland, with very high titres of infectious virus being reported from milk^12,13,16^. However, further mutations that increase polymerase activity in bovine cells are still being reported as the outbreak continues. There have been three separate emergences of PB2 D740N, which we show here, boosting polymerase activity above the wild-type virus. PB2 E627K has also arisen once in conjunction with M631L in a cluster of cattle and appears to be continuing to expand as of July 2025, consistent with this combination giving a robust enhancement in polymerase activity in combination with M631L in our minireplicon assay, and a replicative advantage in cattle mammary explants. It will be vital to continue to monitor the evolution of the polymerase (and other viral genes), and how mutations may impact infectivity and transmissibility of these viruses in cattle and other mammals.

In 2025, the USDA reported two further spillovers of H5N1 into cattle in Nevada and Arizona from the D1.1 genotype^20,28^, which was the predominant genotype in wild birds at the time of spillover. Interestingly, neither cluster has PB2 E627K nor PB2 M631L, despite D1.1 PB2 having >98.5% amino acid identity with the B3.13 PB2 (and similarly arising from low pathogenicity avian influenza virus of North American waterfowl). The Nevada cluster has the known mammalian ANP32 adaptive mutation, PB2 D701N^26^, whereas the Arizona cluster has several mutations in the polymerase relative to the closest avian sequences, which have yet to be tested for mammalian activity, such as PB2 R379K and D678N. These independent emergences provide further evidence that E627K does not have a strong selective advantage to the influenza polymerase in cattle, and that there are many different viable pathways for H5N1 polymerase adaptation in mammals.

What is the current risk to humans posed by the cattle virus? Our results show that the cattle virus containing mammalian adaptations such as PB2 M631L can replicate better in mammalian cells than avian-like viruses, and is therefore a clear risk to mammals that have consumed infected milk, such as cats and raccoons^13^. Thus far, these cattle viruses show inefficient airborne transmissibility in ferret experiments^33,47^ and have not adapted to transmit via the respiratory route or to efficiently use human receptors (α-2,6-linked sialic acids), which would be essential for human-to-human transmission^48–51^. In addition, there are also other potential blocks to emergence posed by antiviral host factors such as MxA to which the cattle virus remains susceptible^52^. Nonetheless, frequent exposure to an antigenically novel, highly pathogenic influenza virus that can replicate well in human cells is concerning. Our data suggest that the emerged cattle influenza virus is also capable of efficiently infecting avian and swine cells, further increasing the risk of spillover from cattle to other species. In fact, the mammalian-adapted virus appeared to have no fitness cost in avian cells, explaining the high propensity of this virus to spillover into poultry, causing multiple outbreaks, and suggesting such a virus could maintain its mammalian adaptations if it spilled back into wild waterfowl. With each human infection and increased polymerase activity leading to higher levels of replication, there is a danger of further evolution changing viral receptor properties. Additionally, a reassortment event with human seasonal influenza viruses could lead to a novel virus, particularly during the Northern Hemisphere winter influenza season.

Recent experimental infections in cows suggest that the ability of the North American B3.13 cattle influenza virus to spread via milk is not unique and that other mammalian viruses are capable of transmitting via this route^16^. Phylogenetic data suggest that this originated from a single spillover event from wild birds to cattle and, despite recent spillovers from D1.1, such spillovers are likely unusual as there is scant evidence of IAV in cattle prior to 2024. Nonetheless, in the absence of an effective control strategy, the highly pathogenic H5N1 virus may now become endemic in US dairy cattle, requiring continuous monitoring even in the absence of overt disease. Moreover, many other clades and strains of the H5N1 virus continue to emerge through reassortment, causing zoonotic infections. Urgent development and testing of broadly reactive H5 influenza vaccines for both animals and humans is a priority.

## Methods

### Ethics and biosafety

Virus work was undertaken at either containment level 3 (CL3), SAPO4 (for whole H5N1 reverse genetics-derived viruses) or CL2 (for reverse genetics-derived viruses with HA and NA from the attenuated vaccine strain A/Puerto Rico/8/1934 (PR8) and the remaining internal genes from H5N1 viruses). Viruses carrying H5 HA with a multibasic cleavage site are categorised as specified animal pathogens order (SAPO) 4 and Advisory Committee on Dangerous Pathogens (ACDP) hazard group 3 by United Kingdom regulations. Work with these viruses was undertaken in a licensed CL3/SAPO4 facility of The Pirbright Institute under GMRA (BAG-RA-226). CL2 work with ACDP Hazard Group 2 recombinant influenza viruses was performed at the Roslin Institute under biological risk assessments BARA 1011 and GMRA 1811. All virus and GM risk assessments were approved by the appropriate internal committees, as well as the UK Health and Safety Executive (HSE) and, where necessary, the UK Scientific Advisory Committee for Genetic Modification (SACGM).

Ethics for the use of the primary human airway epithelial cultures (hNECs) were as described previously^53^. Briefly, donors provided written consent, and Ethics approval was given through the Living Airway Biobank, administered through the UCL Great Ormond Street Institute of Child Health (REC reference: 19/NW/0171, IRAS project ID: 261511, Northwest Liverpool East Research Ethics Committee). Nasal brushings were obtained by trained clinicians from adult (30–50 years) donors who reported no respiratory symptoms in the preceding 7 weeks. Brushings were taken from the inferior nasal concha zone using cytological brushes (Scientific Laboratory Supplies, CYT1050). All methods were performed following the relevant guidelines and regulations.

Generation of bovine explants came under approval from the University of Glasgow School of Biodiversity, One Health and Veterinary Medicine (EA26/25).

### Cells

Human Embryonic Kidney 293 T (293 T), Madin-Darby Canine Kidney (MDCK), MDCK overexpressing chicken ANP32A (MDCK-ggANP32A), chicken immortalised fibroblast cells (DF-1), human adenocarcinoma cells (A549), duck immortalised fibroblasts (CCL-141), human immortalised lung fibroblasts (MRC5), and bovine mammary epithelial cells (MAC-T) were maintained in Dulbecco’s modified Eagle medium (DMEM) supplemented with 10% foetal bovine serum (FBS), and 1% Pen-Strep. MAC-T at Roslin additionally had 2 mM L-glutamine, 5 µg/ml insulin and 1 µg/ml of hydrocortisone. Human lung adenocarcinoma (Calu-3) cells were maintained in DMEM with 10% FCS, 1% Pen-Strep, and 2 mM L-glutamine. Swine foetal testes (ST) cells were maintained in Advanced DMEM, 5% FCS, 1% Pen-Strep, and 2 mM L-glutamine. Chicken lung epithelial cell (CLEC213^54^, kindly gifted by Dr Sascha Trapp) was cultured in DMEM F12 supplemented with 8% FBS, 2 mM glutamine, and 1% Pen-Strep. Triple knockout cells (tKO- Engineered human haploid (eHAP) cells with ANP32A, ANP32B and ANP32E knocked out) were generated as previously described^31^ and maintained in Iscove’s modified Dulbecco’s medium (IMDM), 10% FBS, 1% NEAA, 1% Pen-Strep. All cells were maintained at 37 °C and 5% CO_2_.

Primary human nasal epithelial cultures (hNECs) were generated as described previously^53^. Briefly, basal epithelial cells from nasal brushings were expanded mitotically inactivated 3T3-J2 fibroblasts to passage 1 and cryopreserved. Cells were thawed as required, expanded with 3T3-J2 fibroblasts to passage 2, and seeded onto collagen I–coated, semi-permeable membrane supports (3 × 10^5 cells/6.5 mm, 0.4 µm pore size, Transwell; Corning). Differentiation was performed under air–liquid interface conditions in PneumaCult™-ALI medium for 4 weeks.

Bovine mammary tissue was collected at Sandyford Abattoir from health lactating cattle. Excess tissue was removed, teat and gland cisterns were dissected and transferred to transport media comprising chilled DMEM supplemented with 100 μg/ml Penicillin/ 100 μg/ml streptomycin and 5 μg/ml fungizone. Every 45 min, tissues were transferred to 500 ml of fresh transport media. Inside the class II biological safety cabinet, explants were made using a 5 mm biopsy punch. Biopsies were added to 24-well plates with 500 ul of transport media supplemented with 10% FBS and incubated for 24 h at 37 °C, 5% CO_*2*_ before infection.

### Plasmids

Reverse genetics plasmids of the viruses used in this study were produced as previously described (Table 1)^51,55,56^. Mutants were generated by site directed mutagenesis. pCAGGS expression plasmids of the polymerase subunits were subcloned from reverse genetics plasmids.

**Table 1:** Virus strains, aliases, accessions and uses in this study

Virus strain name	Alias	GISAID ID	Whole virus, 2:6 or minireplicon
A/Puerto Rico/8/1934	PR8		2:6 HA and NA only
A/dairy cattle/Texas/24-008749-001-original/2024 (H5N1)	cattle/Texas	EPI_ISL_19094762	All
A/chicken/England/053052/2021(H5N1)	AIV07	EPI_ISL_9012457	minireplicon only
A/turkey/England/50-92/1991(H5N1)	50-92	EPI_ISL_5888	minireplicon only
A/England/195/2009	Eng195	EPI_ISL_29994	Whole virus and minireplicon only

Construction of the minireplicon luciferase reporter plasmid for use in bovine cells was performed using a previous strategy used for an equivalent swine reporter, pSPOM2-Firefly^40^, with swine polI promoter replaced with 428 bp bovine polI promoter from the bovine genome, bosTau8 chrUn_GJ059828v1 (1165-1592)^57^. Plasmid map available at github.com/Flu1/bovinePolI. *Bos taurus Anp32A* (NCBI reference sequence NM_001195019.1) and *Anp32B (NM_001035074.1)* were ordered as synthetic GeneArt constructs from ThermoFisher Scientific and subcloned into pCAGGS as described previously^58^.

### Viruses

Viruses were rescued using 8 plasmid pHW2000 (or derivatives) sets containing each genome segment from the named virus. HEK 293 T cells were transfected with a mix containing 250 ng of each plasmid using Lipofectamine 2000 according to the manufacturer’s instructions. Media was replaced 24 h post-transfection with serum-free DMEM with 0.14% bovine serum albumin and 1 μg/ml of L-(tosylamido-2-phenyl) ethyl chloromethyl ketone (TPCK)-treated trypsin. Virus-containing supernatants were collected after 48 h, clarified by centrifugation and used to infect MDCK-ggANP32A cells or inoculate 10-day-old embryonated hen’s eggs. Infected MDCK-*Gg*ANP32A were monitored twice daily, and supernatant was harvested, clarified, aliquoted and stored when a clear cytopathic effect was observed (generally between 48 and 72 hpi). Eggs were incubated for 48 h before humane euthanization at 4 °C, after which virus-containing allantoic fluid was collected, clarified and stored at −80 °C before titration by plaque assay.

### Phylogenetics

Dairy cattle sequences submitted prior to 1^st^ December 2024, avian H5N1 sequences from the USA submitted between 1^st^ September 2023 and 31^st^ March 2024, and A/Texas/37/2024 were downloaded from GISAID. Sequences from isolates which contained PB2, PB1, PA, NP, HA, NA, NS1, NS2/NEP, M1 and M2 segments were concatenated. Sequences were aligned using MAFFT v7.490, trimmed using pytrimal (v0.8.4) with a gap threshold of 0.85 to ensure correct residue numbering (total length = 4466 amino acids). Duplicate sequences and sequences with >30 ambiguous residues or gaps were removed. A phylogenetic tree from the cleaned alignment (*n* = 902) was made in IQTree2 (v2.2.2.6) running automatic ModelFinder and the FLAVI + F + I + R2 model^59,60^. Data manipulation and visualisation were carried out using Python 3.12.2 and R 4.4.1. Code and list of GISAID accessions can be found at https://github.com/fernandocapelastegui/Cattle-H5N1-polymerase-adaptation.

### Minireplicon assay

293 T, MAC-T, or eHAP tKO cells were transfected in 24-well plates with Lipofectamine 3000 transfection reagent (Thermo Fisher) with the following amounts of pCAGGS expression plasmids: 40 ng PB2, 40 ng PB1, 20 ng PA, 80 ng NP, 40 ng Renilla luciferase, 80 ng species-specific (human, bovine, swine or chicken) polI vRNA Firefly luciferase and 40 ng ANP32 or empty pCAGGS (where appropriate). DF-1 and ST cells were transfected in 12-well plates using quadruple the plasmid/transfection reagent mixes to those used for the other cells in 24-well plates. Cells were lysed 24 h post-transfection using passive lysis buffer (Promega) and polymerase activity measured using the Dual-luciferase Reporter assay system (Promega) and a FLUOstar Omega plate reader (BMG Labtech). Assays in MAC-T cells were read using Dual-Glo® Luciferase Assay System (Promega) on an Infinite MPlex plate reader (Tecan). Firefly luciferase signal has been normalised to Renilla luciferase signal to give relative luminescence units (RLU).

### Virus replication kinetics

Confluent monolayers of CLEC213, CCL-141, A549, MRC5, MAC-T, BAT II, and Calu-3 cells were washed once with PBS and infected at a multiplicity of infection (MOI) 0.01 with virus diluted in serum-free medium for 1 h at 37 °C. Inoculum was replaced with serum-free medium supplemented with 0.14% BSA and 1 µg/ml TPCK-treated bovine pancreas trypsin. Calu-3 and MAC-T experiments performed with the whole H5N1 virus excluded TPCK trypsin. Supernatants were collected at various times post-infection and stored at −80 °C until infectious titres were determined by plaque assay.

Infection of primary human airway cultures was performed as follows. Before infection, cells were washed with Dulbecco’s phosphate-buffered saline supplemented with calcium and magnesium (DPBS++) to remove mucus and debris. Cells were infected with 200 µl of virus in DPBS++ at an MOI of 0.01 and incubated at 37 °C for 1 h. Inoculum was removed, and cells were washed twice with DPBS + +. Time points were taken by adding 200 µl of DPBS + + and incubating for 10 min at 37 °C before removal and titration. The time course was performed at 37 °C, 5% CO_2_. Time points were taken at 24−, 48−, 72− and 96-h post-infection. Infectious titres were determined by plaque assay on MDCKs.

Infection of explants was performed as follows: individual explants were transferred to 96-well plates and infected with 5000 PFU of virus in 100 µl of serum-free DMEM for 2 h at 37 °C. Explants were transferred to clean 24-well plates, washed 3x with warm PBS and overlayed with 1 ml of DMEM supplemented with 0.14% fraction V BSA and 1 µg/ml TPCK. Time points were collected at the indicated times post-infection.

### Split Luciferase assays

Split luciferase assays were performed in 293 T cells seeded into 48-well plates. 40 ng of PB2, PA, and PB1 tagged with the N-terminus of *Gaussia* luciferase (Gluc1) on its C-terminus (after a GGSGG linker), were co-transfected with ANP32 tagged with the C-terminus of *Gaussia* luciferase (Gluc2) on its C-terminus (after a GGSGG linker), using Lipofectamine 3000 as per the manufacturer’s instructions. 24 h post-transfection, cells were lysed in 60 µl of *Renilla* lysis buffer (Promega), and luciferase activity was measured using a *Renilla* luciferase kit (Promega) on a VANTAstar plate reader (BMG Labtech). Normalised luminescence ratios (NLRs) were calculated by dividing the values of the tagged PB1 and ANP32 wells by the sum of the mean of the control wells, which contained i) untagged PB1 and free Gluc1, and ii) untagged ANP32 and free Gluc2, as described elsewhere^24^.

### Immunoblotting

Transfected cells were lysed in radioimmunoprecipitation (RIPA) buffer (150 mM NaCl, 50 mM TRIS, 0.1% SDS, 1% NP-40, 0.5% sodium deoxycholate, pH 7.4) supplemented with an EDTA-free protease inhibitor mini tablet (Thermo Fisher), incubated on ice for 10 min, then centrifuged for 10 min to remove precipitate. Lysates were then diluted in 4 x Laemmli buffer (Bio-Rad) and loaded onto 4-20% gradient gels to perform SDS-PAGE. Proteins were transferred onto a PVDF membrane by semi-dry transfer. Membranes were blocked in TBS/5% milk for 1 h at room temperature and incubated overnight at 4 °C with the following primary antibodies: mouse anti-FLAG (Sigma, F1804; 1:250 dilution), rabbit anti-PB2 (GeneTex, GTX125926; 1:500 dilution), rabbit anti-PB1 (Genetex, GTX125923, 1:250 dilution), rabbit anti-PA (Genetex, GTX118991; 1:500 dilution), mouse anti-α-Tubulin (Abcam, ab7291; 1:1250 dilution) rabbit anti-*Gaussia* luciferase (Invitrogen, PIPA1181; 1:1000). Following four 10-min TBS/1% Tween20 (TBS-T) washes, membranes were incubated with the following near-infra-red fluorescent secondary antibodies: goat anti-mouse IgG Alexa FluorRTM 680 (abcam; ab175775; 1:10,000 dilution), goat anti-rabbit IRDye 800CW (LI-COR, 926-32211; 1:10,000 dilution) for 45 min at room temperature. After a further four TBST washes and one TBS wash, membranes were imaged using an Odyssey DLx (Li-Cor Biosciences) using the Image Studio Lite software.

### Virus whole genome sequencing

Viral RNA was extracted from the virus inocula used in hNECs and the 72-h time point using the QIAamp viral RNA extraction kit (Qiagen) according to the manufacturer’s instructions. Viral cDNA was synthesised using the Veso cDNA synthesis kit (Thermo) with a mix of UTR-specific primers (TATTCGTCTCACCCAGCAAAAGCAGG and TATTCGTCTCACCCAGCGAAAGCAGG) with cycling conditions of 42 °C for 75 min, 95 °C for 2 min. The Optil-F1 (GTTACGCGCCAGCAAAAGCAGG), Optil-F2 (GTTACGCGCCAGCGAAAGCAGG) and Optil-R1 (GTTACGCGCCAGTAGAAACAAGG) primers were used in a PCR from cDNA with PFU Ultra II DNA polymerase (Agilent). Cycling conditions were 55 °C for 2 min, 98 °C for 2 min. 5 x cycles of 94 °C for 30 s, 44 °C 30 s, 68 °C 3 m 30 s, 35x cycles of 94 °C for 30 s, 57 °C for 30 s, 68 °C 3 m 30 s, a final extension step of 68 °C for 10 m followed by a holding step of 4 °C. The PCR products were then purified using the Agencourt AMPure XP kit per manufacturer’s instructions. Purified amplicons were quantified by Qubit (Thermo) and diluted in nuclease-free water to a final concentration of 0.2 ng/µl. 1 ng total of input nucleic acid was processed using the Illumina Nextera XT kit on a Hamilton NGStar. The resulting libraries were confirmed using the Tapestation 4200 *Agilent) and bead-normalised according to the manufacturer’s specifications before being pooled and diluted to 12.5 pM final concentration with 1% 12.5 pM PhiX (Illumina). The resulting pool was then run on an Illumina MiSeq using a 2 × 250 v2 MiSeq reagent cartridge and flow cell.

Raw fastq sequences were then imported into Geneious Prime software (v2019.2) and trimmed to remove primers and aligned to the whole genome reference sequence (A/dairy cattle/Texas/24-008749-001-original/2024 (H5N1)). Sequences were further analysed using a custom R script available at github.com/Flu1/bovineseq. Sequences from defective interfering genomes were removed, and subsequent mutations were analysed in Geneious Prime. Sequence data is available from https://www.ebi.ac.uk/ena under project PRJEB102111.

### Reporting summary

Further information on research design is available in the Nature Portfolio Reporting Summary linked to this article.

## Supplementary information

Supplementary Information

Reporting summary

Transparent Peer Review file

## Source data

Source data

## Data Availability

Source data are provided with this paper in the Supplementary Information/Source Data file. The sequencing data used in this study are available from https://www.ebi.ac.uk/ena under project PRJEB102111. Protein structure PDB: 8R1J was used to map mutations generated from this study. Source data are provided with this paper.

## Code availability

Code for this study is available at https://github.com/fernandocapelastegui/Cattle-H5N1-polymerase-adaptation and https://github.com/Flu1/bovineseq.
