# Supplementary material for: Polymerase mutations underlie early adaptation of H5N1 influenza virus to dairy cattle and other mammals
Source: Nat Commun. 2026 Jan 16;17:1603. doi: 10.1038/s41467-026-68306-6 (PMC12905184; doi:10.1038/s41467-026-68306-6)
Supplement: Supplementary file 2 — Reporting summary [file 41467_2026_68306_MOESM2_ESM.pdf]

nature portfolio

Corresponding author(s): Thomas Peacock and Daniel Goldhill

Last updated by author(s): Dec 9, 2025

Reporting Summary

Nature Portfolio wishes to improve the reproducibility of the work that we publish. This form provides structure for consistency and transparency in reporting. For further information on Nature Portfolio policies, see our Editorial Policies and the Editorial Policy Checklist.

Statistics

For all statistical analyses, confirm that the following items are present in the figure legend, table legend, main text, or Methods section.

n/a	Confirmed	
☐	☒	The exact sample size (*n*) for each experimental group/condition, given as a discrete number and unit of measurement
☐	☒	A statement on whether measurements were taken from distinct samples or whether the same sample was measured repeatedly
☐	☒	The statistical test(s) used AND whether they are one- or two-sided<br>*Only common tests should be described solely by name; describe more complex techniques in the Methods section.*
☒	☐	A description of all covariates tested
☐	☒	A description of any assumptions or corrections, such as tests of normality and adjustment for multiple comparisons
☐	☒	A full description of the statistical parameters including central tendency (e.g. means) or other basic estimates (e.g. regression coefficient) AND variation (e.g. standard deviation) or associated estimates of uncertainty (e.g. confidence intervals)
☐	☒	For null hypothesis testing, the test statistic (e.g. *F*, *t*, *r*) with confidence intervals, effect sizes, degrees of freedom and *P* value noted<br>*Give P values as exact values whenever suitable.*
☒	☐	For Bayesian analysis, information on the choice of priors and Markov chain Monte Carlo settings
☒	☐	For hierarchical and complex designs, identification of the appropriate level for tests and full reporting of outcomes
☒	☐	Estimates of effect sizes (e.g. Cohen's *d*, Pearson's *r*), indicating how they were calculated

Our web collection on statistics for biologists contains articles on many of the points above.

Software and code

Policy information about availability of computer code

Data collection	Code for this study is available at https://github.com/fernandocapelastegui/Cattle-H5N1-polymerase-adaptation and https://github.com/Flu1/bovineseq.
Data analysis	Geneious Prime software (v2019.2).<br>Data manipulation and visualisation was carried out using Python 3.12.2 and R 4.4.1.<br>MAFFT v7.490 was used to align sequences and sequences were trimmed using pytrimal (v0.8.4). A phylogenetic tree was made in IQTree2 (v2.2.2.6).

For manuscripts utilizing custom algorithms or software that are central to the research but not yet described in published literature, software must be made available to editors and reviewers. We strongly encourage code deposition in a community repository (e.g. GitHub). See the Nature Portfolio guidelines for submitting code & software for further information.

Data

Policy information about availability of data

All manuscripts must include a data availability statement. This statement should provide the following information, where applicable:

- Accession codes, unique identifiers, or web links for publicly available datasets
- A description of any restrictions on data availability
- For clinical datasets or third party data, please ensure that the statement adheres to our policy

Source data are provided with this paper in the Supplementary Information/Source Data file. The sequencing data used in this study are available from https://www.ebi.ac.uk/ena under project PRJEB102111. Protein structure PDB: 8R1J (https://www.rcsb.org/structure/8R1J) was used to map mutations generated from this study.

Research involving human participants, their data, or biological material

Policy information about studies with human participants or human data. See also policy information about sex, gender (identity/presentation), and sexual orientation and race, ethnicity and racism.

Reporting on sex and gender	All Human Airway Epithelial donors used were female.
Reporting on race, ethnicity, or other socially relevant groupings	N/A
Population characteristics	30-50 years.
Recruitment	Adult (30–50years) donors who tested negative for SARS-CoV-2 (within 24–48h of sampling) and reported no respiratory symptoms in the preceding 7weeks.
Ethics oversight	Ethics for the use of the primary human airway epithelial cells were as described previously in https://doi.org/10.1038/s41564-024-01658-1. Briefly, donors provided written consent and Ethics approval was given through the Living Airway Biobank, administered through the UCL Great Ormond Street Institute of Child Health (REC reference: 19/NW/0171, IRAS project ID: 261511, Northwest Liverpool East Research Ethics Committee).

Note that full information on the approval of the study protocol must also be provided in the manuscript.

Field-specific reporting

Please select the one below that is the best fit for your research. If you are not sure, read the appropriate sections before making your selection.

☒ Life sciences ☐ Behavioural & social sciences ☐ Ecological, evolutionary & environmental sciences

For a reference copy of the document with all sections, see nature.com/documents/nr-reporting-summary-flat.pdf

Life sciences study design

All studies must disclose on these points even when the disclosure is negative.

Sample size	No statistical methods were used to predetermine sample size. Experiments were performed with N=3 as that is standard in our field.
Data exclusions	No data were excluded.
Replication	All experiments were repeated 3 times in triplicate. Human airway epithelial cells experiments were repeated from three independent donors.
Randomization	Randomization was not relevant for our study. Molecular biology experiments are not routinely randomized.
Blinding	Blinding was not relevant for our study. Molecular biology experiments are not routinely blinded.

Reporting for specific materials, systems and methods

We require information from authors about some types of materials, experimental systems and methods used in many studies. Here, indicate whether each material, system or method listed is relevant to your study. If you are not sure if a list item applies to your research, read the appropriate section before selecting a response.

Materials & experimental systems

n/a	Involved in the study
☐	☒ Antibodies
☐	☒ Eukaryotic cell lines
☒	☐ Palaeontology and archaeology
☐	☒ Animals and other organisms
☒	☐ Clinical data
☐	☒ Dual use research of concern
☒	☐ Plants

Methods

n/a	Involved in the study
☒	☐ ChIP-seq
☒	☐ Flow cytometry
☒	☐ MRI-based neuroimaging

Antibodies

Antibodies used	mouse anti-FLAG (Sigma, F1804; 1:250 dilution), rabbit anti-PB2 (GeneTex, GTX125926; 1:500 dilution), rabbit anti-PB1 (Genetex, GTX125923, 1:250 dilution), rabbit anti-PA (Genetex, GTX118991; 1:500 dilution), mouse anti-α-Tubulin (Abcam, ab7291; 1:1250 dilution) rabbit anti-Gaussia luciferase (Invitrogen, PIPA1181; 1:1000)<br>goat anti-mouse IgG Alexa FluorRTM 680 (abcam; ab175775; 1:10,000 dilution), goat anti-rabbit IRDye 800CW (LI-COR, 926-32211; 1:10,000 dilution)
Validation	Validated as per manufacturer's websites (https://www.sigmaaldrich.com/GB/en/product/sigma/f1804, https://www.genetex.com/Product/Detail/Influenza-A-virus-PB2-protein-antibody/GTX125926, https://www.genetex.com/Product/Detail/Influenza-A-virus-PB2-protein-antibody/GTX125923, https://www.genetex.com/Product/Detail/Influenza-A-virus-PB2-protein-antibody/GTX118991, https://www.abcam.com/en-us/products/primary-antibodies/alpha-tubulin-antibody-dm1a-loading-control-ab7291, https://www.fishersci.com/shop/products/gaussia-luciferase-polyclonal-antibody/PIPA1181 ,https://www.abcam.com/en-us/products/secondary-antibodies/goat-mouse-igg-h-l-alexa-fluor-680-ab175775, https://shop.licorbio.com/irdye-secondary-antibodies/irdye-800cw-goat-anti-rabbit-igg-secondary-antibody/.

Eukaryotic cell lines

Policy information about cell lines and Sex and Gender in Research

Cell line source(s)	293T, MDCK, DF-1, A549, Calu-3, and ST cells were from the ATCC.<br>MRC5 were a kind gift from Dr Finn Grey, University of Edinburgh, UK<br>CCL-141 cells were a kind gift from Dr Leah Golding of University of Nottingham.<br>MDCK-ggANP32 cells were a kind gift from Professor Massimo Palmarini of the Centre for Virological Research, Glasgow.<br>MAC-T cells were a kind gift from Professor J Ross Fitzgerald (Roslin Institute).<br>CLEC213 were a kind gift from Dr Sasha Trapp.<br>3T3-J2 fibroblasts were originally sourced from Simon Broad, Prof. Fiona Watt (King's College London, UK), Dr. Paola Bonfanti (University College London, UK), and Prof. Howard Green (Harvard Medical School, Boston, MA, USA).<br>tKO were derived from eHAP cells from Horizon Discovery as detailed in Sheppard et al. Nat Comms 2023.
Authentication	Species identities of the CLEC213, MAC-T and BAT-II cell lines were confirmed by targetted gene sequencing. Other cells were not specifically authenticated for use in this study.
Mycoplasma contamination	Cells were tested for, and tested negative for mycoplasma contamination. Cells were tested by Venor GEM Advance PCR test (3t3-J2, HEK 293T, DF-1, MDCK cells, MAC-T, CLEC213, NHDF, BAT II, CCL-141, MRC5, A549, eHAPs) or Invivogen Mycostrips (ST cells).
Commonly misidentified lines<br>(See ICLAC register)	*Name any commonly misidentified cell lines used in the study and provide a rationale for their use.*

Animals and other research organisms

Policy information about studies involving animals; ARRIVE guidelines recommended for reporting animal research, and Sex and Gender in Research

Laboratory animals	n/a
Wild animals	n/a
Reporting on sex	All animals were female as this work is specific to cattle mammary glands which are only found in female animals
Field-collected samples	*For laboratory work with field-collected samples, describe all relevant parameters such as housing, maintenance, temperature, photoperiod and end-of-experiment protocol OR state that the study did not involve samples collected from the field.*
Ethics oversight	Generation of bovine explants came under approval from the University of Glasgow School of Biodiversity, One Health and Veterinary Medicine (EA26/25).

Note that full information on the approval of the study protocol must also be provided in the manuscript.

Dual use research of concern

Policy information about dual use research of concern

Hazards

Could the accidental, deliberate or reckless misuse of agents or technologies generated in the work, or the application of information presented in the manuscript, pose a threat to:

No	Yes	
☐	☒	Public health
☒	☐	National security
☐	☒	Crops and/or livestock
☐	☒	Ecosystems
☒	☐	Any other significant area

Hazards: This work describes how an avian influenza virus adapted from wild birds to farmed cattle. This work could potentially be misused to adapt avian influenza viruses to better infect mammals which then might have an impact on public health, mammalian livestock (such as cattle) and wild mammals.

For examples of agents subject to oversight, see the United States Government Policy for Institutional Oversight of Life Sciences Dual Use Research of Concern.

Experiments of concern

Does the work involve any of these experiments of concern:

No	Yes	
☒	☐	Demonstrate how to render a vaccine ineffective
☒	☐	Confer resistance to therapeutically useful antibiotics or antiviral agents
☒	☐	Enhance the virulence of a pathogen or render a nonpathogen virulent
☒	☐	Increase transmissibility of a pathogen
☐	☒	Alter the host range of a pathogen
☒	☐	Enable evasion of diagnostic/detection modalities
☒	☐	Enable the weaponization of a biological agent or toxin
☒	☐	Any other potentially harmful combination of experiments and agents

Precautions and benefits

Biosecurity precautions: The work through this manuscript was either performed generating naturally occuring viruses or mutants, or using a loss of function approach - starting with a virus that already exists in nature with a concerning property (in this case ability to replicate efficiently in mammalian cells) and removing mutations to attenuate it in those systems.

Biosecurity oversight: Virus work was undertaken at either containment level 3 (CL3), SAPO4 (for whole reverse genetics-derived viruses) or CL2 (for reverse genetics-derived viruses with HA and NA from the attenuated vaccine strain A/Puerto Rico/8/1934 (PR8) and the remaining internal genes from H5N1 viruses). Viruses carrying H5 HA with a multibasic cleavage site are categorised as specified animal pathogens order (SAPO) 4 and Advisory Committee on Dangerous Pathogens (ACDP) hazard group 3 by United Kingdom regulations. Work with these viruses was undertaken in a licensed CL3/SAPO4 facility of The Pirbright Institute under GMRA (BAG-RA-226). CL2 work with ACDP Hazard Group 2 recombinant influenza viruses was performed at the Roslin Institute under biological risk assessment BARA 1011 approved by a University of Edinburgh biosafety committee and GMRA 1811. All virus and GM risk assessments were approved by the appropriate internal committees, as well as the UK Health and Safety Executive (HSE) and, where necessary, the UK scientific advisory committee for genetic modification (SACGM).

Benefits: This work helps us understand how the cattle H5N1 viruses have adapted to Cattle during the outbreak in the USA. This is useful for surveillance efforts in the current (and any future) outbreak, risk assessing the threat to humans and other mammals and mechanistically understanding how avian influenza viruses can cross the species barrier into mammals, as they have several times in the past few years.

Communication benefits: Although this work could pose an information hazard, mutations of the type described here have long been described in the literature (as we state transparently through the manuscript). Therefore we believe this information helps surveillance and risk assessment with very little information hazard.

Plants

Seed stocks	*Report on the source of all seed stocks or other plant material used. If applicable, state the seed stock centre and catalogue number. If plant specimens were collected from the field, describe the collection location, date and sampling procedures.*
Novel plant genotypes	*Describe the methods by which all novel plant genotypes were produced. This includes those generated by transgenic approaches, gene editing, chemical/radiation-based mutagenesis and hybridization. For transgenic lines, describe the transformation method, the number of independent lines analyzed and the generation upon which experiments were performed. For gene-edited lines, describe the editor used, the endogenous sequence targeted for editing, the targeting guide RNA sequence (if applicable) and how the editor was applied.*
Authentication	*Describe any authentication procedures for each seed stock used or novel genotype generated. Describe any experiments used to assess the effect of a mutation and, where applicable, how potential secondary effects (e.g. second site T-DNA insertions, mosiacism, off-target gene editing) were examined.*
